# Vasoconstrictor effect of Africanized honeybee (*Apis mellifera L.*) venom on rat aorta

**DOI:** 10.1186/1678-9199-19-24

**Published:** 2013-09-25

**Authors:** Paulo César P Sousa, Teresinha S Brito, Daniel S Freire, Rafael M Ximenes, Pedro Jorge C Magalhães, Helena SA Monteiro, Renata S Alves, Alice Maria C Martins, Daniela O Toyama, Marcos H Toyama

**Affiliations:** 1 Department of Physiology and Pharmacology, Federal University of Ceará, Fortaleza, Ceará State, Brazil; 2 Department of Clinical and Toxicological Analyses, Federal University of Ceará, Fortaleza, Ceará State, Brazil; 3 Center of Biological and Health Sciences, Mackenzie Presbyterian University, São Paulo, São Paulo State, Brazil; 4 Paulista Coast Experimental Campus, São Paulo State University (UNESP – Univ Estadual Paulista), São Vicente, São Paulo State, Brazil; 5 Department of Antibiotics, Federal University of Pernambuco, Recife, Pernambuco State, Brazil

**Keywords:** *Apis mellifera*, Venom, Mellitin, Phospholipase A_2_, Aorta

## Abstract

**Background:**

*Apis mellifera* stings are a problem for public health worldwide, particularly in Latin America due to the aggressiveness of its Africanized honeybees. Massive poisoning by *A. mellifera* venom (AmV) affects mainly the cardiovascular system, and several works have described its actions on heart muscle. Nevertheless, no work on the pharmacological action mechanisms of the AmV in isolated aorta has been reported. Thus, the present work aimed to investigate the actions of AmV and its main fractions, phospholipase A_2_ (PLA_2_) and melittin, on isolated aorta rings and a probable action mechanism.

**Results:**

AmV and the complex PLA_2_ + melittin (0.1-50 μg/mL) caused contraction in endothelium-containing aorta rings, but neither isolated PLA_2_ nor melittin were able to reproduce the effect. Endothelium removal did not change the maximum vasoconstrictor effect elicited by AmV. Ca^2+^-free medium, as well as treatment with phentolamine (5 μM), verapamil (10 μM), losartan (100 μM), and U-73122 (10 μM, a phospholipase C inhibitor), eliminated the AmV-induced contractile effects.

**Conclusions:**

In conclusion, AmV caused contractile effect in aorta rings probably through the involvement of voltage-operated calcium channels, AT1 and α-adrenergic receptors via the downstream activation of phospholipase C. The protein complex, PLA_2_ + melittin, was also able to induce vasoconstriction, whereas the isolated proteins were not.

## Background

Africanized honeybees are a poly-hybrid species found in South America, mainly in Brazil, known for their aggressiveness and involvement in mass attacks [1,2]. The clinical manifestations of bee envenomation are characterized by local and systemic toxicity. Among the systemic manifestations observed after bee stings are cardiovascular changes and renal damage. Despite the significant advances made in understanding the pathogenesis of nephrotoxic and ischemic acute renal failure caused by such envenomation, there have been few changes in terms of mortality [3].

*A. mellifera* venom (AmV) is a complex mixture of enzymes, peptides and amines. Among the components of the venom are biogenic amines, hyaluronidases, phospholipases A_2_ (PLA_2_) and melittin, which is its main constituent, approximately 50% of the dry weight [4,5]. This active 26-amino-acid peptide is released from its precursor, promelittin, during biosynthesis and presents hemolytic activity. It also is the primary allergen found in bee venom [6]. It acts synergistically with phospholipase A_2_ (PLA_2_) to enhance its toxic actions [7].

Despite published *in vivo* characterization of the cardiovascular effects of both melittin and PLA_2_, few *in vitro* experiments that clarify the action mode of these toxins are available in the scientific literature [8]. Some papers report the role of endothelium, lipoxygenase products, and nitric oxide (NO) in the vascular effects of melittin [9-11]. The present study aimed to investigate the vasoconstrictor effect of crude *A. mellifera* venom (AmV) and its fractions PLA_2_, melittin, and the native linked fraction (PLA_2_ + melittin), on isolated aortic rings of rats.

## Methods

### Venom and reagents

*A. mellifera* venom was purchased from Bio-Agents Serpentarium (Brazil). All chemicals and solvents were of analytical grade and purchased from certified suppliers (Bayer, Sigma-Aldrich, Labtest Diagnostic and Applied Biosystems).

### Animals

Male Wistar rats (250–300 g) were maintained under standard conditions of temperature and humidity in 12-hour light–dark cycles. The animals had fasted for 24 hours before any experimental procedure, and water was provided *ad libitum*. All experiments were in accordance with the guidelines for the ethical use of experimental animals published by the Brazilian College on Experimental Animal Care (COBEA), with a project license approved by the Animal Ethics Committee of Federal University of Ceará (protocol number 01/2012).

### Purification of PLA_2_ and melittin from *Apis mellifera* venom

The bee venom PLA_2_ was purified in two chromatographic steps. Briefly, AmV was dissolved (10 mg/mL) in NaCl 0.9% and kept at 14°C under constant stirring. The protein content of the venom was precipitated with pure acetone for 24 hours, centrifuged (4500 x *g*) and lyophilized to obtain the protein fraction, which was used for the fractionation of PLA_2_. In the first step, the protein fraction was subjected to a molecular exclusion chromatography, using a size exclusion column (1 x 60 cm) previously packed with Superdex®75 (GE Healthcare Life Sciences, USA). The column was attached to semi-preparative HPLC and equilibrated with a 0.2M ammonium bicarbonate buffer (pH 7.8). The protein fraction (15 mg) was dissolved in 250 μL of the buffer, homogenized, and centrifuged at 4500 x g for five minutes. The supernatant was filtered through a 0.45 μm filter and then injected into the column. Fractionation of the venom was performed at a constant flow rate of 0.3 mL/minute and monitored at 280 nm. Each fraction was collected for three minutes (0.9 mL) and tested for phospholipase A_2_ enzymatic activity.

After confirming the PLA_2_ enzymatic activity, the fraction was subjected to a new chromatographic step on reversed-phase HPLC using a C18 semi-analytical column, which was equilibrated with buffer A (0.1% TFA), to isolate the PLA_2_ and melittin. Samples of the fraction with PLA_2_ activity (3 mg) were dissolved in 250 mL of buffer A, clarified and injected into the chromatographic column. The elution was performed with a continuous linear gradient of buffer B (66% acetonitrile in 0.1% TFA) and the monitoring was done at 280nm. The samples from the first chromatographic step were analyzed by SDS-PAGE, using a running gel of 12% acrylamide and a stacking gel of 5% acrylamide, with bisacrylamide (0.32%) as cross-linker.

### Mass spectrometry

The molecular masses of the PLA_2_ and melittin were determined by matrix-assisted laser desorption/ionization time-of-flight (MALDI-TOF) mass spectrometry using a Voyager-DE PROMALDI-TOF mass spectrometer (Applied Biosystems®, Life Technologies™, USA). One microliter of the sample dissolved in 0.1% TFA was mixed with 2 μL of the matrix (α-cyano-4-hydroxycinnamic acid). The matrix was prepared with 30% acetonitrile and 0.1% TFA (v/v). Ion masses were determined at an acceleration voltage of 25 kV, the laser operating at 2890 μJ/com2, with a 300 ns delay and a linear analysis mode.

### Aortic ring assay

The rats were sacrificed by stunning followed by cervical dislocation. The thoracic aorta were removed and immersed in a perfusion medium (Krebs-Henseleit solution) at room temperature. After removing adhering fat and connective tissue, each aorta was cut transversally into cylindrical ring-like segments (1 x 5 mm) attached to steel wire triangular pieces (0.3 mm diameter), which were suspended in a 5 mL organ bath containing Krebs-Henseleit solution (pH 7.4) with the following composition (mM): NaCl 118.0, KCl 4.7, MgSO4 1.2, CaCl2 2.5, KH2PO4 1.2, NaHCO3 25.0 and glucose 11.1, continuously aerated at 37°C with 95% O_2_ and 5% CO_2_.

In some aortic tissues, endothelium was removed immediately after dissection by gentle rubbing of the aortic lumen with a stainless steel wire. Endothelium-containing or denuded strips were stretched with a passive tension of 1 g while the tension was recorded using an isometric force transducer (ML870B60/C-V, AD Instruments, Australia) connected to a digital data acquisition system (PowerLab™ 8/30, AD Instruments). After an equilibration period of at least 60 minutes, control contractions were induced by adding a submaximal concentration (60 mM) of potassium chloride (KCl) to the bath. When two successive control contractions showed similar amplitudes, preparations were considered equilibrated. Contraction data were expressed as percentages of the potassium-induced contraction. Endothelium-containing or -denuded preparations were contracted at the beginning of the experiment with potassium (30 mM) and after establishment of a contractile plateau, they were challenged with 1 μM of acetylcholine. The lack of acetylcholine-induced vasorelaxant effects was interpreted as evidence that the preparation was effectively denuded of endothelium [3].

### Effects of the *A. mellifera* venom (AmV) in isolated rat aorta

Concentration-effect curves of AmV were obtained by exposing a preparation in basal tonus to cumulatively increasing concentrations of AmV (0.1 to 50 μg/mL) added to the bath and maintaining it at a given concentration for five minutes. The vasoconstrictor effects of AmV were determined in preparations without functional endothelium. In order to investigate the vasoconstrictor effect of AmV, concentration-effect curves were constructed by exposing the preparations to cumulatively increasing concentrations of AmV in basal tonus in preparations maintained in a Ca^2+^-containing medium, in a Ca^2+^-free medium (containing 2 x 10^-5^ M EGTA), and in the presence of verapamil (10 μM), a well-known L-type voltage-operated Ca^2+^ channel (VOCC) blocker, phentolamine (5 μM), an α-adrenergic blocker, losartan (100 μM), an angiotensin II AT1 receptor antagonist, and U73122 (10 μM), a phospholipase C inhibitor.

### Effects of the AmV fractions, PLA_2_ and melittin, on isolated rat aorta

In order to investigate whether the fractions PLA_2_ and melittin were involved in the vasoconstrictor effects of the AmV, concentration-effect curves (0.1 to 50 μg/mL) were constructed by exposing the preparations to cumulatively increasing concentrations of the fractions, PLA_2_, melittin and the complex PLA_2_ + melittin (1:1, w:w).

### Statistical analysis

Data were reported as means ± SEM. The means were evaluated by ANOVA, followed by the Holm-Sidak *post hoc* test. Emax values were compared using the unpaired Student’s *t* test. The acceptance level for statistically significant differences was set at 5% (p < 0.05).

## Results

### Fractionation of *A. mellifera* crude venom

**Figure 1:**
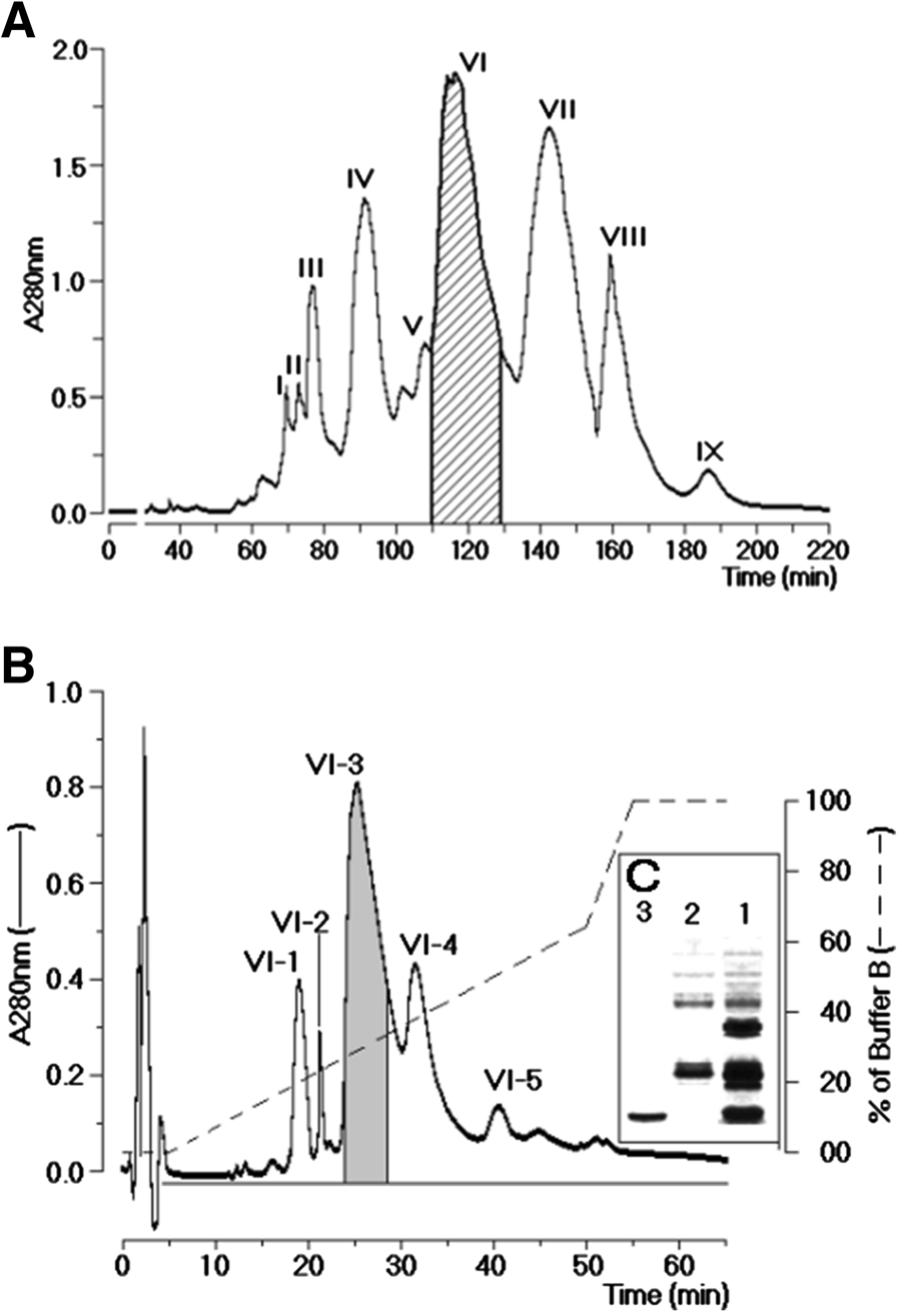
**Fractionation of AmV. (A)** Chromatography of whole protein extract from honeybee venom using a molecular exclusion column packed with Superdex®75. The chromatographic run was carried out at a flow rate of 0.6 mL/h and monitored at 280 nm. **(B)** Reverse phase HPLC run yielded five main fractions designated from VI-1 to VI-5. Fraction VI-3 was confirmed as PLA_2_ by specific phospholipase A_2_ assay and fraction VI-4 as melittin after MALDI-TOFF analysis. **(C)** SDS-PAGE analysis of the purified proteins.

*A. mellifera* crude venom fractionation through size exclusion chromatography showed the presence of nine fractions, designated sequentially I to IX, as shown in Figure 1A. Only the fraction VI showed phospholipasic A_2_ enzymatic activity. The SDS-PAGE analysis of fraction VI revealed the presence of two major fractions as well as a minor electrophoretic band (results not shown). This fraction was subjected to a reverse phase HPLC. The chromatographic profile of the purification of the fraction VI showed five peaks, which were denominated sequentially VI-1 to VI-5, where the most important fractions in terms of chromatogram area were VI-3 and VI-4 (Figure 1B). The analysis on SDS-PAGE of these fractions showed that VI-3 had a molecular mass of approximately 15 kDa (Figure 1C). Fraction VI-4 was not detected using a 10% acrylamide gel. This result suggested that fraction VI-4 must have a molecular mass lower than 10 kDa. PLA_2_ enzymatic activity was found only in fraction VI-3, whereas fraction VI-4 reduced the PLA_2_ of fraction VI-3. Finally, a MALDI-TOF analysis, to confirm the molecular homogeneity of PLA_2_ and melittin, revealed respective molecular masses of 15,343.31 Da and 3,101.03 Da for the PLA_2_ and melittin.

### Effect of *A. mellifera* venom and its fractions in isolated aorta

**Figure 2:**
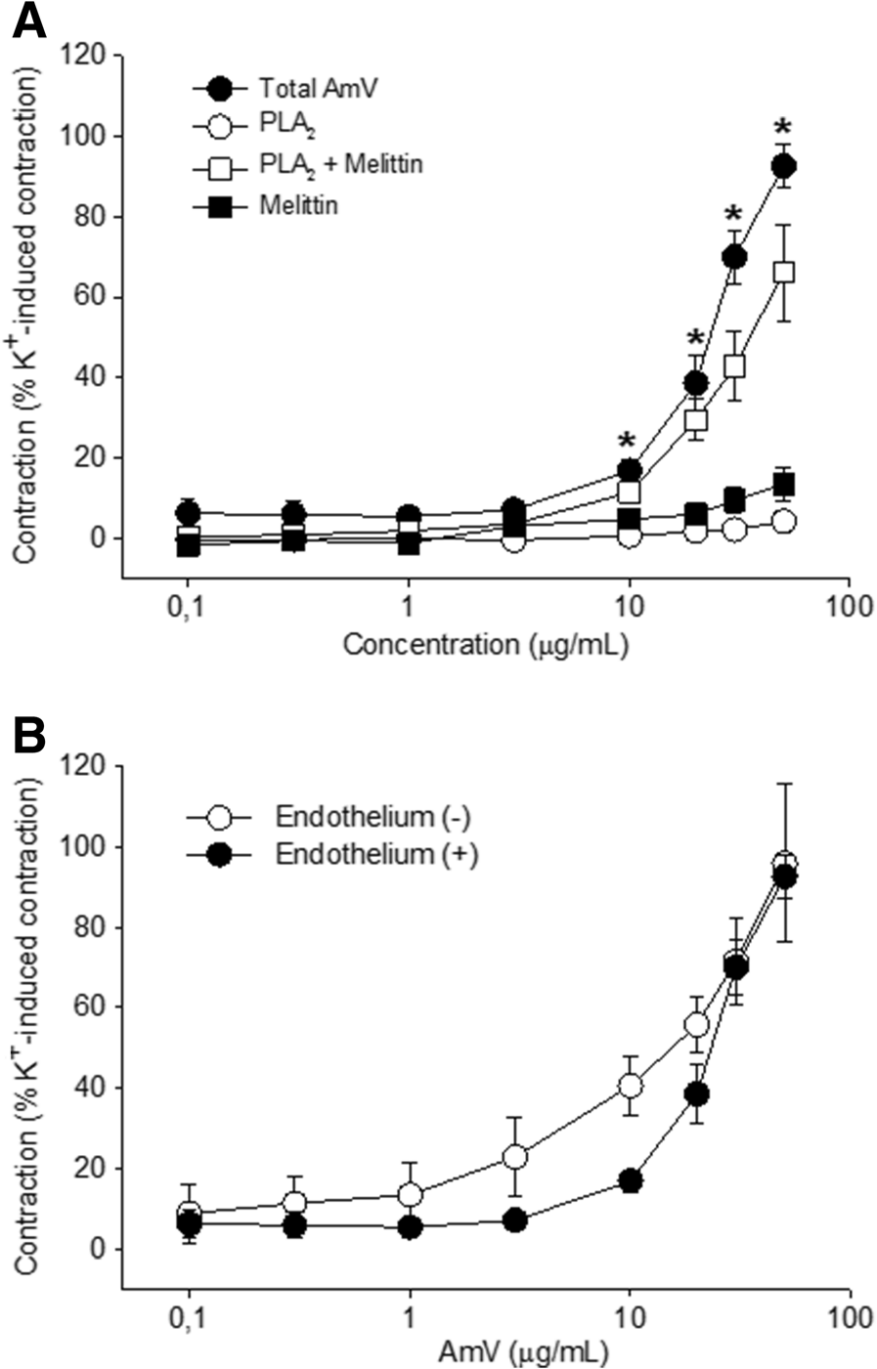
**Characterization of the vasoconstrictor effect of AmV. (A)** Vasoconstrictor effect of AmV (0.1-50 μg/mL) (●, n = 5) and the fractions: melittin (■, n = 5); PLA_2_ (Ο, n = 5); and the complex PLA_2_ + melittin (□, n = 5). **(B)** Effect of endothelium removal on AmV vasoconstrictor effect. The concentration response curve of AmV (0.1-50 μg/mL) on basal tone in endothelium-containing aorta preparations (●, n = 5), and in endothelium-denuded aorta preparations (Ο, n = 4). Vasoconstrictor effects are expressed as a percentage of the contractile response to K^+^ (60 mM). Data are expressed as mean ± SEM and analyzed by ANOVA followed by the Holm-Sidak *post hoc* test.

In endothelium-containing aorta preparations, AmV (0.1-50 μg/mL) induced contractile responses in a concentration-dependent manner. The magnitude of the contraction induced by 50 μg/mL of AmV corresponded to 92.6 ± 5.5% (n = 5 rings) of a reference contraction induced by 60 mM K^+^. In the same preparation, the isolated PLA_2_ or melittin (0.1-50 μg/mL) did not induce significant contractile responses. However, the complex PLA_2_ + melittin (0.1-50 μg/mL) induced a vasoconstrictor effect in a concentration-dependent manner, (n = 4 rings; p < 0.05; Figure 2A). The magnitude of the contractile effect at 50 μg/mL was 66.1 ± 11.9% of the contraction induced by K^+^ (60 mM). Endothelium removal did not change the maximum vasoconstrictor effect elicited by AmV (Figure 2B).

### Elucidation of the mode of action of *A. mellifera* venom in isolated aorta

**Figure 3:**
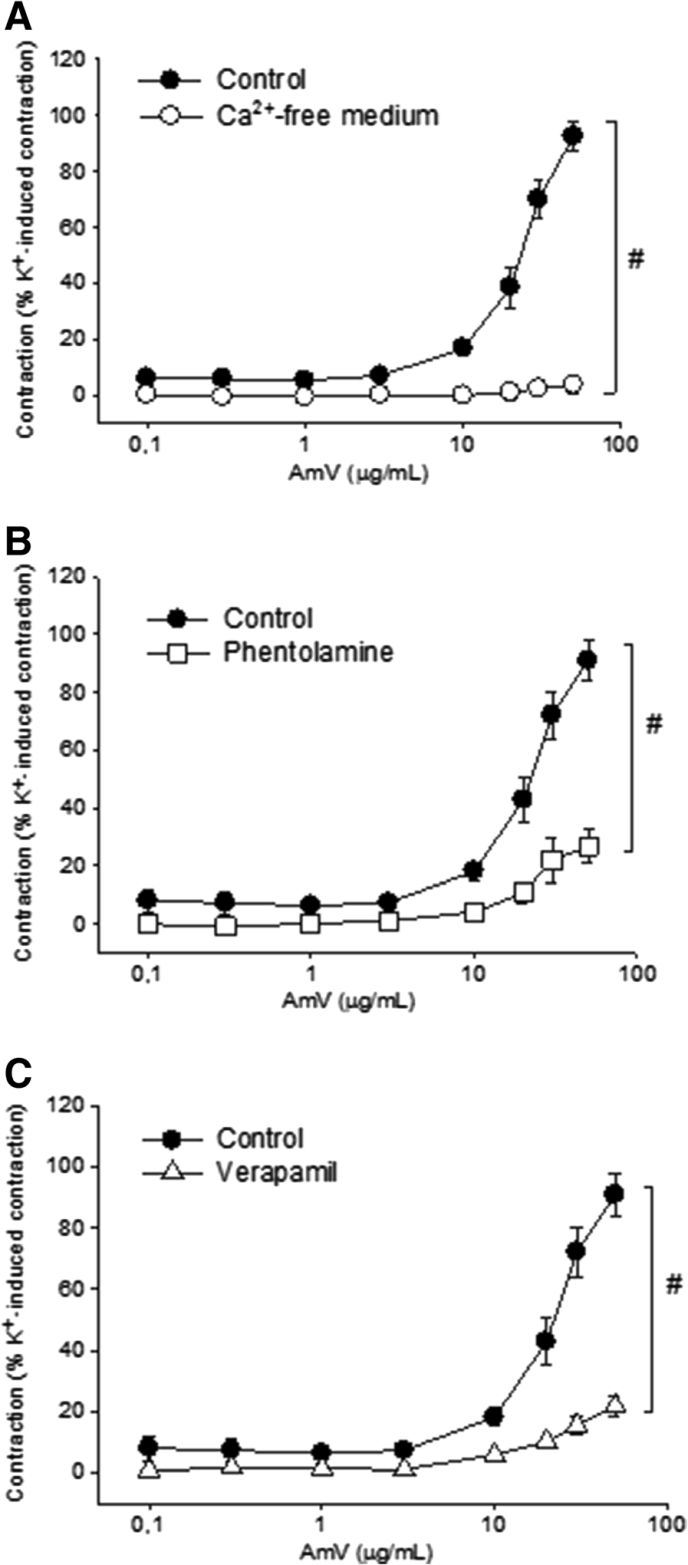
**Study on the probable action mode of AmV.** Vasoconstrictor effects of AmV: **(A)** Ca^2+^-free medium (O, n = 5); **(B)** pretreatment with phentolamine (5 μM; □, n = 5); **(C)** pretreatment with verapamil (10 μM; Δ, n = 5). Vasoconstrictor effects are expressed as a percentage of the contractile response to K^+^ (60 mM). Data are expressed as mean ± SEM and analyzed by ANOVA followed by the Holm-Sidak posttest.

In aortic rings maintained in the Ca^2+^-free medium, the contractile effects induced by AmV were abolished and the magnitude of the maximal contraction was 3.7 ± 3.0% of the K^+^ (60 mM) value (n = 5; p < 0.05; Figure 3A), a value significantly lower than that observed in Ca^2+^-containing medium. Pretreatment of aortic rings with phentolamine (5 μM) also significantly reduced AmV -induced contraction (50 μg/mL) to 26.8 ± 5.6% of the K^+^ (60 mM) value (n = 5 rings; p < 0.05; Figure 3B). In order to evaluate the effect of a voltage-gated calcium channel blocker on AmV-induced contraction, preparations were treated with verapamil (10 μM) after which the contractions elicited by AmV (50 μg/mL) significantly diminished to 21.7 ± 3.3% of the K^+^ (60 mM) value (n = 4 rings; p < 0.05; Figure 3C).

**Figure 4:**
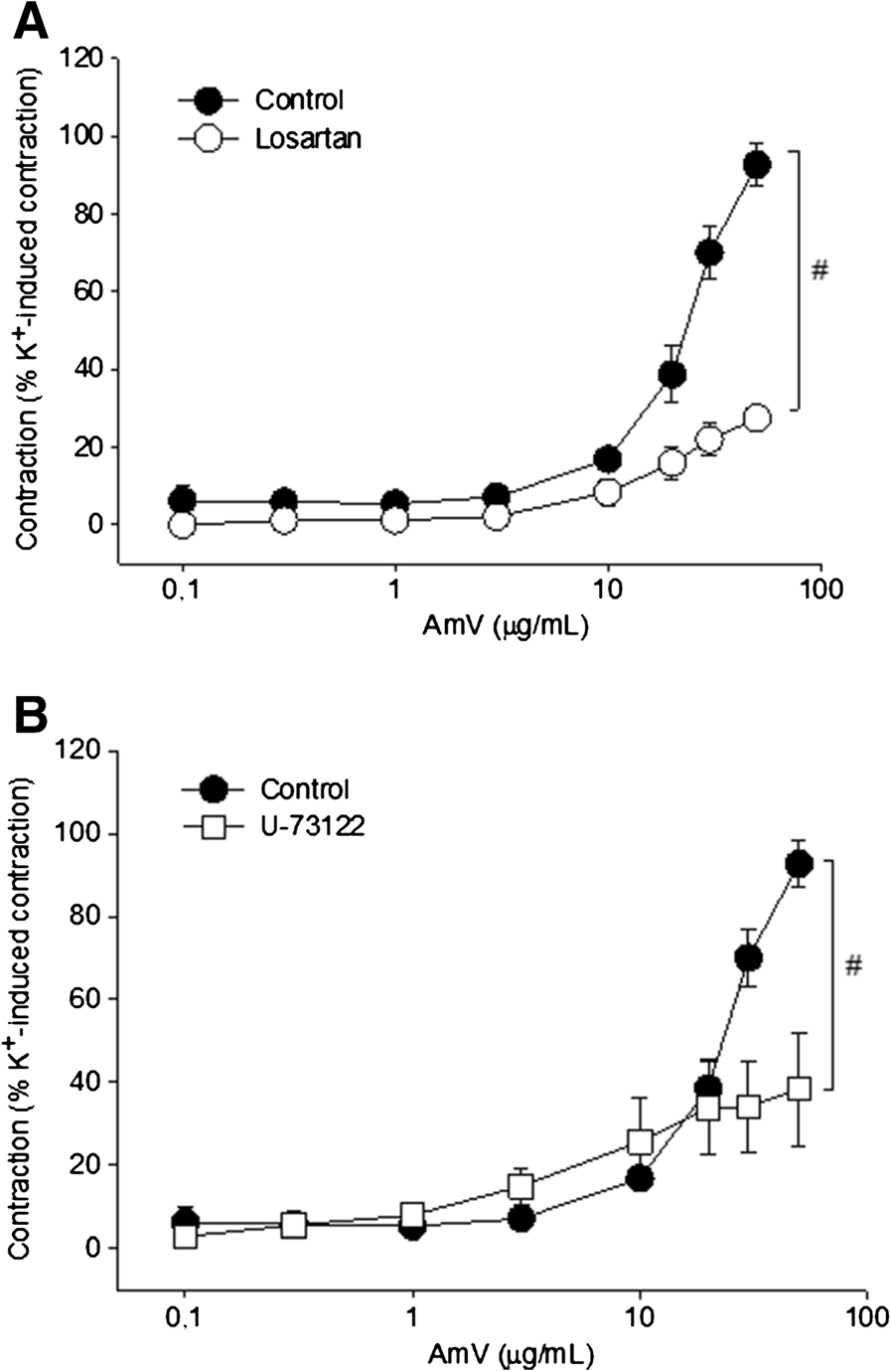
**Study on the probable action mode of AmV.** Vasoconstrictor effects of AmV: **(A)** pretreatment with losartan (100 μM; O, n = 5); **(B)** pretreatment with U-73122 (10 μM; □, n = 5). Vasoconstrictor effects are expressed as a percentage of the contractile response to K^+^ (60 mM). Data are expressed as mean ± SEM and analyzed by ANOVA followed by the Holm-Sidak posttest.

Aiming to verify whether angiotensin was involved in the contractions induced by AmV, an AT1 receptor antagonist was employed. The preparations were treated with losartan (100 μM), and the contractions elicited by AmV were decreased to 27.3 ± 2.4% of the K^+^ (60 mM) value (n = 4 rings; p < 0.05; Figure 4A). Moreover, pretreatment of aortic rings with U-73122 (10 μM), a phospholipase C inhibitor, significantly reduced AmV-induced contraction (50 μg/mL) to 38.4 ± 5.6% of the K^+^ (60mM) value, respectively (n = 5 rings; p < 0.05; Figure 4B).

## Discussion

The present work describes the isolation of three fractions from the venom of *A. mellifera*, a mixed fraction of PLA_2_ and melittin, and both in pure protein form PLA_2_ and melittin. These proteins together represent approximately 60% of the venom dry weight [12,13]. A vasoconstrictor effect of AmV and the complex PLA_2_ + melittin was confirmed. However, the isolated PLA_2_ and melittin had no contractile effect on the aortic rings. Some authors reported that melittin facilitates the interaction of PLA_2_ with the cell membrane, exposing the membrane to the catalytic site of the enzyme due to its amphipathic characteristics [3]. In order to investigate the action mode of *A. mellifera*-induced vasoconstriction, the involvements of extracellular Ca^2+^, voltage-gated calcium channels, α_2_-adrenergic receptors, AT1 receptors and phospholipase C were assayed.

In Ca^2+^-free medium, an inhibition of the contractile response of AmV was observed. Likewise, employing verapamil, a calcium channel blocker, produced an absence of the AmV-induced contractile response. We suggested that the vasoconstrictor properties are dependent on a trans-membrane Ca^2+^ influx. Thus, the opening of voltage-operated calcium channels is involved in the mechanism proposed for the contractile effect of AmV.

In most cases, a smooth muscle contraction involves a combination of input and Ca^2+^ release. The various types of smooth muscle may differ markedly in this regard: in the relative contribution of the two sources of Ca^2+^ for contraction (intracellular and extracellular) and in ion channels through which extracellular Ca^2+^ can have access to the interior of the cell [14]. According to Jackson [15], the contraction of vascular smooth muscle occurs primarily by the influx of Ca^2+^ into the muscle cell. If the extracellular concentration of Ca^2+^ is reduced, the contraction is almost eliminated.

Adrenergic receptors consist of typical G-protein-coupled receptors. Each of these receptor classes, α_1_- and α_2_-adrenergic receptors, is associated with a specific system of secondary messengers. The α_1_-adrenergic receptors are coupled to phospholipase C and produce their effects mainly through the release of intracellular Ca^2+^ stores, mediating vasoconstriction [16].

In an attempt to elucidate the pathway involved in the contraction of the aorta by AmV, an α-adrenergic receptor blocker (phentolamine), an AT1 receptor antagonist (losartan), and a phospholipase C inhibitor (U-73122) were used. A significant reduction in the contractile effect of AmV on the aortic rings was observed, suggesting the involvement of these receptors in tissue contraction via phospholipase C.

This hypothesis is corroborated by Vinhote *et al*. [17], who reported such contraction in aortic rings, induced by *Polybia paulista* wasp venom, showing that the effect was dependent on voltage-operated calcium channels, and that α-adrenergic receptors were involved.

## Conclusions

In conclusion, *Apis mellifera* venom causes a contractile effect on aorta rings probably through the involvement of voltage-operated calcium channels, AT1 and α-adrenergic receptors via the downstream activation of phospholipase C. In contrast to the isolated proteins, the protein complex PLA_2_ + melittin was also able to induce vasoconstriction.

### Ethics committee approval

The present study was approved by the Animal Ethics Committee of Federal University of Ceará (protocol number 01/2012). Moreover, all experiments were in accordance with the guidelines for the ethical use of experimental animals published by the Brazilian College on Experimental Animal Care (COBEA).

## Competing interests

The authors declare that there are no competing interests.

## Authors’ contributions

PCPS, TSB, DSF, RSA, DOT and performed the biochemical and pharmacological experiments. PJCM, HSAM, AMCM, MHT designed the study and discussed the results. RMX, AMCM and MHT drafted the manuscript. All authors read and approved the manuscript.
